# Screening Key Genes for Salt Tolerance in Maize Inbred Lines via Time-Series Transcriptomics and Machine Learning

**DOI:** 10.3390/plants15162480

**Published:** 2026-08-16

**Authors:** Tongwen Shang, Xiaomei Zhang, Lu Tian, Yuan Li, Dongqing Zhang, Youqiang Li, Kaiyue Liu, Shuzhe Wang, Zhaobin Chen, Yajie Zhao, Shaowei Yu, Xiangyu Zhao, Chao Zhou

**Affiliations:** 1 School of Life Sciences, Shandong Agricultural University, Tai’an 271018, China; shangtongwen@126.com (T.S.); 19560857539@163.com (X.Z.); tianlu8211@163.com (L.T.); 13519314547@163.com (Y.L.); 17860832242@163.com (D.Z.); youqiangli2020@163.com (Y.L.); a15206872179@163.com (K.L.); wangsz20010708@163.com (S.W.); cczb246@163.com (Z.C.); 198591yy@163.com (Y.Z.); yusw@sdau.edu.cn (S.Y.); 2 National Center of Technology Innovation for Comprehensive Utilization of Saline-Alkali Land, Dongying 257347, China

**Keywords:** *Zea mays*, salt tolerance, time-series transcriptomics, phenotypic analyses, germplasm evaluation

## Abstract

A systematic evaluation of salt tolerance at the seedling stage was conducted using 143 maize inbred lines under a 150 mM mixed-salt solution (NaCl:Na_2_SO_4_ = 9:1, EC = 16.78 dS/m) that mirrors the ionic composition of saline groundwater in the Yellow River Delta. The comprehensive salt tolerance index (D value) ranged from 0.15 to 0.85 across the population, with the elite line B114 exhibiting the highest D value (0.835) and the sensitive line PHT55 ranking near the bottom. Under salt stress, B114 displayed remarkable growth stability, with plant height decreasing by only 25.9%, fresh weight by 13.3%, and dry weight remaining unchanged, whereas PHT55 suffered severe growth inhibition (plant height: 61.5% decrease; fresh weight: 63.2% decrease; dry weight: 33.3% decrease). Time-series RNA-seq of root tissues across four time points (5, 8, 11, and 14 days) revealed markedly distinct transcriptional dynamics: B114 exhibited relatively stable temporal regulation (2261–9124 DEGs), whereas PHT55 showed a pronounced early transcriptional burst that progressively intensified (3728–10,108 DEGs). Using random forest-based machine learning, 50 core salt tolerance-related genes were unbiasedly identified from 16,194 significantly differentially expressed genes. Functional enrichment analysis revealed that these genes were primarily involved in redox regulation, ion homeostasis maintenance, and stress signal transduction pathways. qRT-PCR validation confirmed biphasic expression patterns, with Zm00001d024160 showing the strongest early induction (48-fold at 5 h). This study established a maize salt tolerance evaluation system closely aligned with field conditions and demonstrated that coordinated temporal transcriptional regulation represents a core molecular mechanism underlying high salt tolerance in maize. The elite salt-tolerant germplasm and key candidate genes identified here provide valuable genetic resources and a theoretical foundation for molecular breeding of salt-tolerant maize adapted to saline-alkaline soils.

## 1. Introduction

Soil salinization is one of the major abiotic stresses limiting global agricultural sustainability and threatening food security [1]. Globally, saline and sodic soils cover approximately 1.381 billion hectares, accounting for about 10.7% of the total land area [2,3]. Coastal saline-alkali regions, as key reserve arable land resources worldwide, represent critical areas for future increases in global food production [4].

Maize is one of the most important cereal crops worldwide, with a global planting area exceeding 197 million hectares and an annual production of over 1.2 billion tons, and plays a central role in feed supply, industrial processing, and global food security [5,6]. Major maize-producing regions are distributed across North America, South America, Europe, and Asia, and these intensive production systems constitute the core of global maize supply [7]. However, in coastal saline-alkali regions, severe soil salinization markedly impairs maize germination and seedling establishment, resulting in weak growth and significant yield reduction, thereby severely constraining regional agricultural development [8,9]. These coastal saline-alkali regions worldwide are generally characterized by shallow groundwater tables (0.5–2.0 m), high salinity (1–30 g/L), and pronounced seasonal salt accumulation [10,11]. The soils in these regions are mainly dominated by chloride-sulfate mixed salts, with chloride (Cl^−^) and sulfate (SO_4_^2−^) as the major anions and sodium (Na^+^) as the dominant cation, which fundamentally differs from the single NaCl stress conditions commonly used in the laboratory [12,13,14,15].

From the perspective of utilization structure, maize consumption is primarily feed-oriented, with industrial use as a secondary component [16]. Feed consumption accounts for approximately 70.5% of total maize utilization and underpins the global livestock industry, whereas industrial consumption represents 24.6%, mainly for starch and fuel ethanol production. Food and specialty uses account for less than 5% of total consumption. The diversified and continuously expanding market demand has intensified the global need for developing salt-tolerant and high-yielding maize cultivars [17,18,19].

Salt stress severely inhibits crop growth and yield formation through three major mechanisms: osmotic stress, ion toxicity, and oxidative damage [20,21,22]. High salinity conditions restrict root water uptake, thereby inducing physiological drought [23,24]. Excessive accumulation of sodium (Na^+^) and chloride (Cl^−^) ions disrupts cellular ion homeostasis and suppresses enzyme activity and photosynthetic efficiency [25,26,27]. Meanwhile, excessive reactive oxygen species (ROS) accumulation triggers membrane lipid peroxidation, leading to cellular membrane damage and impaired nutrient absorption, ultimately resulting in reduced germination, weakened growth, and substantial declines in biomass and yield [28,29,30,31]. Maize is considered a moderately salt-tolerant crop, but the seedling stage is particularly sensitive to salt stress. Salt stress can reduce plant height and biomass accumulation during the seedling stage by 30–70% [32,33,34]. Globally, maize yield losses in moderately saline soils generally range from 30% to 50%, whereas severe salinity conditions may even result in complete crop failure [9].

However, current research and evaluation systems for maize salt tolerance still exhibit several critical limitations, which have severely constrained the screening of salt-tolerant germplasm and the progress of molecular design breeding for salt-tolerant maize [35,36]. First, with respect to salt stress simulation systems, the vast majority of studies employ single NaCl treatments to mimic salinity stress, which fails to accurately represent the mixed-salt environments commonly found in saline-alkali regions worldwide [12,13,14,15]. As a result, substantial discrepancies often arise between laboratory findings and field performance [37]. Second, existing evaluation methods remain relatively simplistic, relying primarily on a limited number of static morphological traits and lacking comprehensive, multi-trait, and dynamic assessment systems capable of fully characterizing salt tolerance [38,39,40,41]. Moreover, transcriptomic studies are still relatively limited, as most analyses are based on single time-point measurements, making it difficult to capture the dynamic regulatory patterns of gene expression under salt stress and to elucidate the differential responses between salt-tolerant and salt-sensitive genotypes [42,43,44]. In addition, current strategies for candidate gene identification are generally inefficient and heavily dependent on subjective judgment, which may overlook regulatory genes with subtle but biologically important changes, thereby hindering the unbiased discovery of salt tolerance-related genes from high-dimensional transcriptomic datasets [45,46].

In recent years, time-series transcriptomics and machine learning have provided promising approaches for overcoming the aforementioned limitations. Time-series transcriptomics enables precise characterization of dynamic gene expression patterns, while machine learning algorithms, such as random forest, allow the objective identification of key feature genes from high-dimensional omics datasets [47,48]. These approaches have already demonstrated substantial potential in studies of crop stress resistance [46,49]. In this study, the Yellow River Delta of China was selected as a representative coastal saline-alkali region for investigation, and a chloride-dominated mixed-salt stress system matching the local groundwater ionic composition (NaCl:Na_2_SO_4_ = 9:1) was established [4,10,11]. Using this system, 143 maize inbred lines were systematically evaluated for salt tolerance at the seedling stage to identify elite salt-tolerant germplasm [38,40]. Furthermore, the salt-tolerant inbred line B114 and the salt-sensitive inbred line PHT55 were selected for integrated analyses combining physiological phenotyping, time-series transcriptomics, and random forest-based machine learning [50]. The aims of this study were to identify core salt tolerance-related genes via time-series transcriptomics and machine learning and to establish a salt tolerance evaluation system that is more closely aligned with field conditions, thereby providing new theoretical insights, elite germplasm resources, and candidate genes for salt-tolerant maize breeding [18,19,51].

## 2. Results

### 2.1. A Novel Screening System for Salt-Tolerant Maize Inbred Lines

In germplasm resource screening, the establishment of precise salt solution systems represents a critical step that substantially influences the scientific validity, accuracy, and reliability of the screening results [4,10].

Comparative analyses showed that a mixed-salt solution containing NaCl and Na_2_SO_4_ at a molar ratio of 9:1 closely matched the physicochemical characteristics of local groundwater [13,14]. The groundwater in Dongying was characterized by high electrical conductivity (47.61–48.42 ms/cm) and high salinity (2.85–2.98%), with Cl^−^ and Na^+^ as the predominant ions and trace amounts of SO_4_^2−^ likely present. The 9:1 mixed-salt solution, in which NaCl accounted for 90% of the total salt composition, ensured consistency with the dominant ionic composition of the local groundwater, whereas the addition of a small proportion of Na_2_SO_4_ simulated the presence of sulfate ions in natural groundwater. The specific salt composition and concentration ratios are provided in Supplementary Table S1.

The mixed-salt solution exhibited a pH value of 7.43, representing a neutral to slightly alkaline environment that closely matched the pH of the groundwater (7.24–7.52). This condition more accurately reflected the natural saline environment than the strongly alkaline 150 mM Na_2_CO_3_ solution (EC = 19.61 dS/m, pH = 11.64) or relatively acidic single-salt solutions, such as 150 mM NaCl (EC = 14.69 dS/m, pH = 6.98), thereby minimizing acid-base interference and enhancing the physiological relevance of the screening results. In this study, 143 maize inbred lines representing diverse germplasm resources were subjected to mixed-salt stress treatment for salt tolerance evaluation and screening. During the seedling-stage assessment, mixed-salt stress significantly inhibited increases in plant height and root length across the maize inbred lines, while fresh weight and dry weight were reduced to varying degrees. Notably, substantial variation in salt tolerance was observed among different inbred lines. Statistical analyses of growth-related morphological traits, including plant height, root length, fresh weight, dry weight, and fresh weight-to-dry weight ratio, for the 143 maize inbred lines are presented in Supplementary Table S2.

The results revealed substantial variation among the 143 maize inbred lines across multiple morphological traits. Under control conditions, the coefficients of variation (CVs) for these traits ranged from 20.89% to 36.76%, whereas under mixed-salt stress conditions, the CVs increased to 25.44–40.66%. Specifically, under mixed-salt stress, the CV values for the evaluated traits were 38.09%, 40.66%, 39.14%, 25.44%, and 28.27%, respectively.

### 2.2. Correlation and Phenotypic Analyses of Salt Tolerance in Maize Inbred Lines

**Figure 1:**
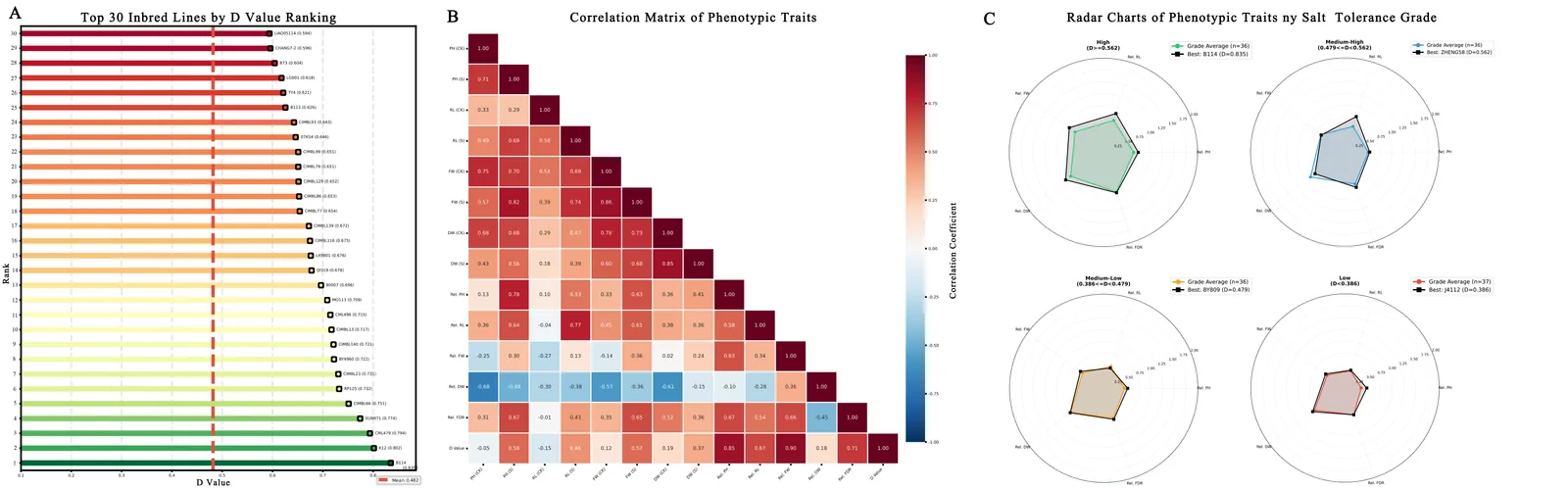
Comprehensive salt tolerance evaluation and correlation analysis of phenotypic and physiological traits in maize inbred lines. (**A**) Bar plot of the top 30 maize inbred lines ranked by the comprehensive salt tolerance index (D value). Bars are color-coded by D value magnitude (green: low; red: high); the red dashed vertical line indicates the population mean D value threshold. (**B**) Pearson correlation matrix heatmap of 13 phenotypic and physiological traits measured under salt stress (n = 143 inbred lines). Color intensity indicates the magnitude and direction of correlation coefficients (dark red: strong positive; dark blue: strong negative). Trait abbreviations: PH, plant height; RWC, relative water content; SOD, superoxide dismutase; POD, peroxidase; CAT, catalase; MDA, malondialdehyde; H_2_O_2_, hydrogen peroxide; Pro, proline; SS, soluble sugars; SP, soluble protein; Na^+^, sodium content; K^+^, potassium content; Na^+^/K^+^, sodium-to-potassium ratio. (**C**) Radar charts comparing normalized trait values (PH, RWC, SOD, POD, CAT) across four salt tolerance grades determined by D value quartiles of 143 maize inbred lines: High (D ≥ 0.562, n = 4), Medium-High (0.479 ≤ D < 0.562, n = 5), Medium-Low (0.386 ≤ D < 0.479, n = 5), and Low (D < 0.386, n = 5). Solid shaded polygons represent the grade average; dashed outlines represent the best-performing genotype within each grade, with genotype name and D value annotated. The D value was calculated using the membership function method: D = (μ_1_ + μ_2_ + μ_3_)/3, where μ_1_, μ_2_, and μ_3_ represent the membership function values for relative plant height, relative root length, and relative biomass, respectively.

Based on the comprehensive salt tolerance index, three highly salt-tolerant inbred lines, B114 (D = 0.835), K12 (D = 0.802), and CML479 (D = 0.794), were further identified (Figure 1A). Heatmap analysis further showed that these inbred lines maintained relatively high plant height, root length, and dry weight under salt stress, indicating strong adaptability and biomass accumulation capacity under saline conditions. The salt tolerance of the maize inbred lines exhibited a clear gradient distribution, further reflecting the abundant genetic variation in salt tolerance among the germplasm resources. These elite germplasm lines provide valuable genetic materials for subsequent studies on the molecular mechanisms of salt tolerance.

To further elucidate the intrinsic relationships between phenotypic traits and the comprehensive salt tolerance index (D value), a correlation matrix heatmap was constructed using all measured traits (Figure 1B; Supplementary Table S3). Strong positive correlations were observed between the absolute traits under control (CK) and salt stress (S) conditions. For example, plant height (PH) exhibited a correlation coefficient of r = 0.71 between CK and S conditions, whereas fresh weight (FW) showed an even stronger correlation (r = 0.86). These results indicate that inbred lines exhibiting superior growth performance under normal conditions were generally able to maintain relatively favorable growth under salt stress, reflecting stable growth potential under adverse conditions. More importantly, the comprehensive D value showed strong positive correlations with all relative traits, including relative plant height (Rel. PH, r = 0.85), relative root length (Rel. RL, r = 0.67), relative fresh weight (Rel. FW, r = 0.90), and relative fresh weight-to-dry weight ratio (Rel. FDR, r = 0.71). These findings demonstrate that relative growth-retention traits under salt stress are the major contributors to the D value, thereby validating the scientific robustness and reliability of the comprehensive salt tolerance evaluation system.

Notably, relative dry weight (Rel. DW) exhibited significant negative correlations with several absolute traits measured under both CK and salt stress conditions, such as plant height under CK (r = −0.68) and salt stress conditions (r = −0.48). These results suggest that inbred lines with higher relative dry weight generally possessed lower basal growth performance but exhibited a stronger capacity to maintain dry matter accumulation under salt stress, implying the presence of distinct underlying salt tolerance mechanisms in these genotypes. In addition, significant synergistic correlations were observed among relative traits. For instance, relative plant height (Rel. PH) was positively correlated with relative root length (Rel. RL) (r = 0.58), indicating coordinated growth responses between aboveground and belowground tissues under salt stress.

To further characterize the phenotypic differences among maize inbred lines with different salt tolerance levels, radar plots of relative phenotypic traits were constructed (Figure 1C). Inbred lines classified as highly salt-tolerant (High, D ≥ 0.562) exhibited strong tolerance across all evaluated traits, with most average relative values approaching or exceeding the baseline value of 1.0, indicating minimal growth inhibition under salt stress. Among these traits, relative dry weight and relative fresh weight-to-dry weight ratio displayed the highest average values. Notably, the elite salt-tolerant line B114 (D = 0.835) showed trait values substantially exceeding 1.0, reflecting superior dry matter accumulation capacity and stress resistance. Inbred lines belonging to the medium-high salt tolerance group (Medium-High, 0.479 ≤ D < 0.562) exhibited relatively balanced performance, with average trait values slightly lower than those of the highly salt-tolerant group but still close to the 1.0 baseline. Relative plant height and relative root length were the dominant traits in this group, suggesting that these genotypes were able to maintain both shoot and root growth under salt stress.

In contrast, inbred lines classified as medium-low salt tolerance (Medium-Low, 0.386 ≤ D < 0.479) and low salt tolerance (Low, D < 0.386) exhibited pronounced growth inhibition, with most relative trait values falling below 0.75. The low salt tolerance group displayed the smallest radar coverage area, indicating severe suppression of growth under salt stress. Even the best-performing line within this group, BY809 (D = 0.479), failed to exceed the baseline value of 1.0, further confirming its limited salt tolerance capacity.

**Figure 2:**
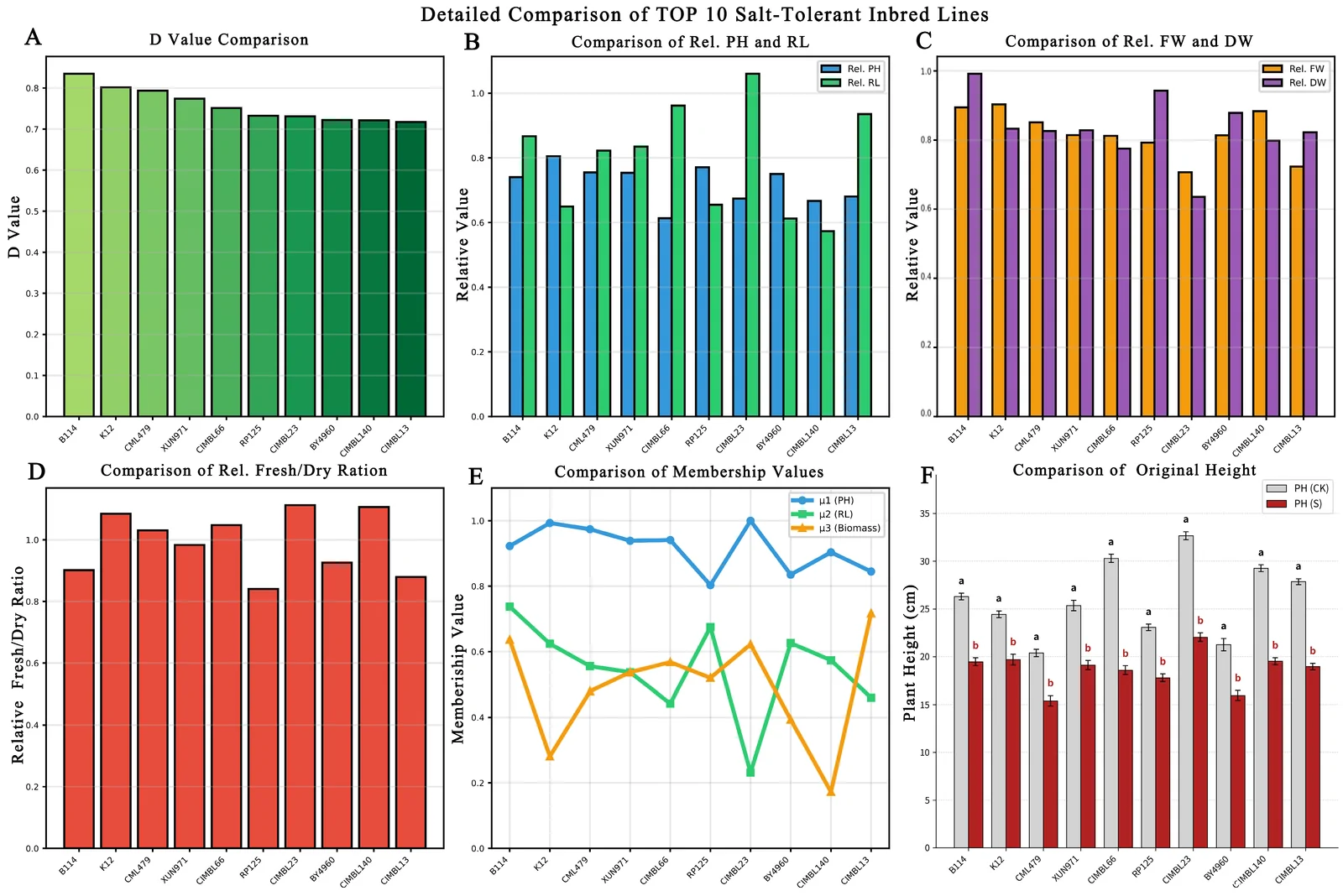
Comprehensive salt tolerance evaluation of the top 10 salt-tolerant maize inbred lines. (**A**) Bar plot comparing the comprehensive salt tolerance index (D value) of the top 10 salt-tolerant maize inbred lines, with bars color-coded by D value. (**B**) Grouped bar plot comparing the relative values (Salt/CK) of plant height (Rel. PH) and root length (Rel. RL). (**C**) Grouped bar plot comparing the relative values of fresh weight (Rel. FW) and dry weight (Rel. DW). (**D**) Bar plot comparing the relative fresh weight-to-dry weight ratio (Rel. FDR). (**E**) Line plot showing the membership function values for plant height (μ_1_), root length (μ_2_), and biomass (μ_3_) across the top 10 inbred lines. (**F**) Grouped bar plot comparing original plant height under control (CK, gray) and salt stress (S, red) conditions. Error bars represent standard deviation (SD; n ≥ 5). Different lowercase letters above the bars indicate significant differences between CK and salt stress treatments within each genotype (Student’s *t*-test, *p* < 0.05). Rel. = Relative; PH = Plant Height; RL = Root Length; FW = Fresh Weight; DW = Dry Weight; FDR = Fresh/Dry Ratio; CK = Control; S = Salt stress.

Scatter plots combined with linear regression analyses were constructed to further assess the contributions of individual phenotypic traits to the comprehensive salt tolerance index (D value) (Supplementary Figure S1A). The D value exhibited strong positive correlations with three core traits, including relative fresh weight (Rel. FW, r = 0.896) (Supplementary Figure S1A), relative plant height (Rel. PH, r = 0.852) (Supplementary Figure S1A), and relative fresh weight-to-dry weight ratio (Rel. FDR, r = 0.706) (Supplementary Figure S1A). These results indicate that the retention of shoot biomass and plant height under salt stress is the major driving factor contributing to high D values and represents the most critical indicator for evaluating salt tolerance in maize. The D value also showed a moderate positive correlation with relative root length (Rel. RL, r = 0.668) (Supplementary Figure S1A), suggesting that root growth maintenance contributes to salt tolerance, although its contribution is lower than that of aboveground traits. Notably, relative dry weight displayed only a weak positive correlation with the D value (r = 0.178), indicating that dry matter retention under salt stress is not a major determinant of the comprehensive D value. Therefore, inbred lines with high D values do not necessarily exhibit high relative dry weight, reflecting the existence of distinct salt tolerance strategies among different genotypes. Figure 2E further demonstrated the negative relationship between salt tolerance ranking and D value, visually illustrating the continuous gradient distribution of salt tolerance among the maize inbred lines. The D values ranged from 0.15 to 0.85, further confirming the robustness and reliability of the evaluation system. Collectively, these findings clarify the quantitative relationships between individual phenotypic traits and the comprehensive salt tolerance index, thereby providing a solid theoretical basis for identifying key selection indicators in maize salt tolerance breeding.

### 2.3. Phenotypic Analysis and Clustering of Salt Tolerance in Maize Inbred Lines

To further validate the reliability of the membership function-based comprehensive salt tolerance evaluation system, the distributions and interrelationships of three key membership function values (μ_1_, μ_2_, and μ_3_) were analyzed. Histogram analyses showed that all three membership values exhibited unimodal and approximately normal distributions, with mean values ranging from 0.47 to 0.50 (Supplementary Figure S1B). Specifically, the mean values were 0.483 for plant height membership (μ_1_), 0.470 for root length membership (μ_2_), and 0.504 for biomass membership (μ_3_), the latter being the highest among the three. These concentrated distribution patterns indicate that the membership values are well suited for comprehensive evaluation and that the overall salt tolerance level of the population is moderate, reflecting substantial genetic diversity for salt tolerance within the tested germplasm.

Scatter plot analysis revealed a significant positive relationship between μ_1_ and μ_2_ (Supplementary Figure S1B), indicating that inbred lines with high plant height membership values generally also exhibited high root length membership values. More importantly, data points with high D values (represented by yellow-to-green colors, indicating strong salt tolerance) were predominantly clustered in the upper-right region of the plot, where both μ_1_ and μ_2_ were high. In addition, these points were characterized by larger marker sizes, representing higher biomass membership values (μ_3_). These results demonstrate that the comprehensive salt tolerance index (D value) is jointly determined by the coordinated contributions of the three membership values. Inbred lines exhibiting high membership values for plant height, root length, and biomass consistently displayed superior salt tolerance, further supporting the robustness and scientific validity of the membership function-based evaluation system. Notably, biomass membership (μ_3_) exhibited the highest average value among the three membership parameters, suggesting that biomass-related traits are major contributors to salt tolerance.

To further elucidate the phenotypic basis underlying salt tolerance, the top 10 inbred lines ranked by D value were selected for detailed multi-trait comparative analyses. The D value ranking plot (Figure 2A) confirmed the superiority of these elite lines, with B114 exhibiting the highest D value (approximately 0.85), followed by K12, CML479, XUN971, and others. All top 10 inbred lines displayed D values greater than 0.70, substantially exceeding the population mean (0.482), thereby confirming their outstanding salt tolerance. Analysis of relative traits revealed the existence of distinct salt tolerance strategies among these highly tolerant inbred lines. For relative plant height and relative root length (Figure 2B), B114 exhibited particularly high relative plant height, whereas CIMBL23 showed exceptionally high relative root length (approximately 1.05), suggesting that some genotypes primarily maintain shoot growth under salt stress, while others rely on sustained root development to enhance stress adaptation. For relative fresh weight and relative dry weight (Figure 2C), all top 10 inbred lines exhibited relatively high values, with most exceeding 0.70. This observation is consistent with the strong positive correlation previously identified between relative fresh weight and the D value. Similarly, the relative fresh weight-to-dry weight ratio (Figure 2D) exceeded 0.80 in all top-performing lines, with most values approaching 1.0, indicating remarkable stability of biomass allocation under salt stress. This characteristic appears to be a key feature associated with superior salt tolerance. The membership function profiles (Figure 2E) further demonstrated that the three membership values (μ_1_, μ_2_, and μ_3_) remained consistently high across the top 10 inbred lines, indicating that high D values resulted from the coordinated contributions of plant height-, root length-, and biomass-related traits. These findings further validate the reliability of the membership function-based evaluation system. Analysis of absolute plant height (Figure 2F) showed that high-D-value inbred lines maintained significantly greater plant height under both control and salt stress conditions, suggesting that superior intrinsic growth potential constitutes an important foundation for salt tolerance.

### 2.4. Phenotypic Divergence Between Salt-Tolerant and Salt-Sensitive Maize Inbred Lines

To validate the reliability of the population-level salt tolerance evaluation system, two maize inbred lines with contrasting salt tolerance phenotypes were selected for detailed phenotypic and physiological analyses under salt stress (Supplementary Table S4): the highly salt-tolerant line B114 and the salt-sensitive line PHT55.

**Figure 3:**
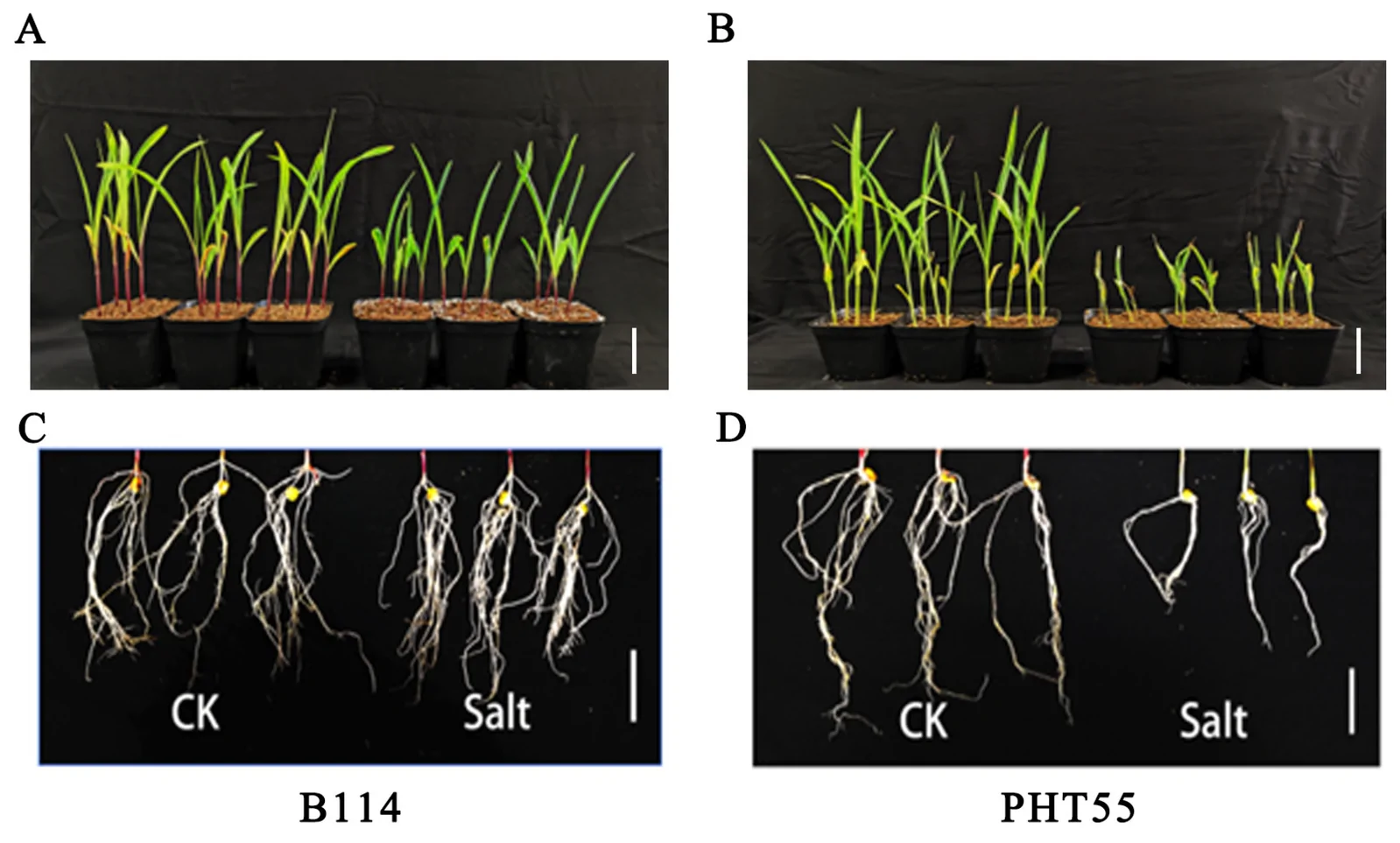
Representative growth phenotype photographs of B114 and PHT55 seedlings under control (CK) and salt stress (Salt) treatments. (**A**) Aboveground phenotype of B114 under control (**left**) and salt stress (**right**) treatments. (**B**) Aboveground phenotype of PHT55 under control (**left**) and salt stress (**right**) treatments. (**C**) Root phenotype of B114 under control (**left**) and salt stress (**right**) treatments. (**D**) Root phenotype of PHT55 under control (**left**) and salt stress (**right**) treatments. Salt stress was imposed using 150 mM mixed-salt solution (NaCl:Na_2_SO_4_ = 9:1) for 14 days. Scale bar = 5 cm.

Under control conditions, both genotypes exhibited normal growth, characterized by vigorous shoot development and well-developed root systems. However, pronounced differences in morphological responses emerged following salt stress treatment. The highly salt-tolerant line B114 showed minimal growth inhibition, maintaining robust shoot growth with only a slight reduction in plant height. Its root system remained largely intact, with abundant lateral roots and limited visible damage under salt stress. In contrast, the salt-sensitive line PHT55 exhibited severe growth suppression, including a substantial reduction in plant height, leaf wilting and chlorosis, and impaired root development characterized by shortened primary roots and a marked reduction in lateral root formation (Figure 3A–D).

**Figure 4:**
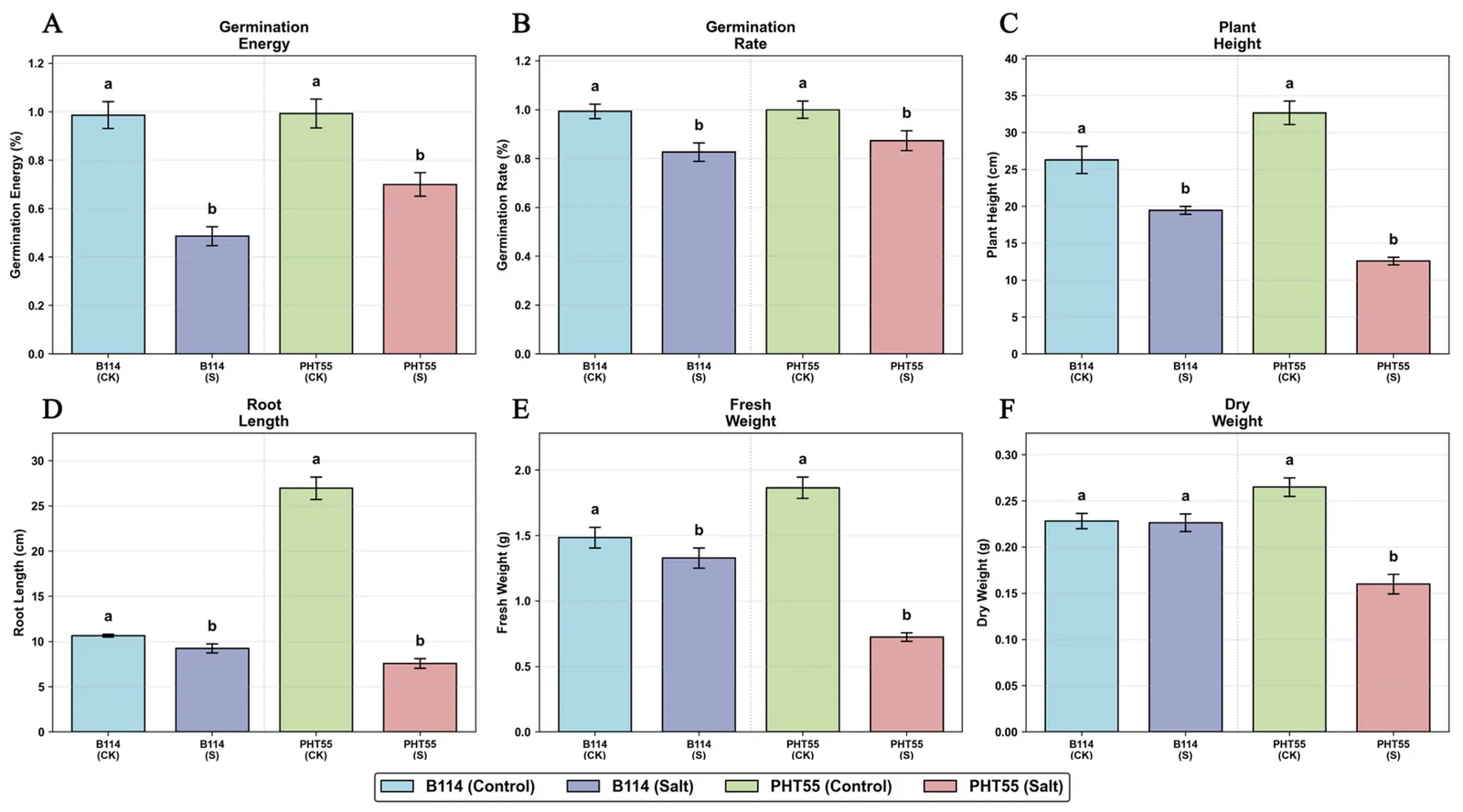
Growth phenotype and physiological trait differences between maize inbred lines B114 and PHT55 under control (CK) and salt stress treatments. (**A**) Germination energy of B114 and PHT55 under control and salt stress treatments. (**B**) Germination rate of B114 and PHT55 under control and salt stress treatments. (**C**) Plant height of B114 and PHT55 under control and salt stress treatments. (**D**) Root length of B114 and PHT55 under control and salt stress treatments. (**E**) Fresh weight of B114 and PHT55 under control and salt stress treatments. (**F**) Dry weight of B114 and PHT55 under control and salt stress treatments. For all panels, gray bars represent the control (CK), and red bars represent salt stress (150 mM mixed-salt solution, NaCl:Na_2_SO_4_ = 9:1, 14 days). Error bars represent standard deviation (SD; n ≥ 5 seedlings per replicate, with ≥10 biological replicates). Different lowercase letters above the bars indicate significant differences between CK and salt stress treatments within each genotype (Student’s *t*-test, *p* < 0.05).

Both B114 and PHT55 exhibited 100% germination vigor and germination rate under control conditions, indicating no significant difference in seed viability between the two genotypes. Following salt stress treatment, germination-related traits declined in both lines (Figure 4A,B). The germination vigor of B114 decreased from 1.0 to 0.5, and its germination rate decreased from 1.0 to 0.8, whereas PHT55 showed a reduction in germination vigor from 1.0 to 0.7 and in germination rate from 1.0 to 0.9. These results suggest that salt stress exerted a stronger inhibitory effect on seed germination in B114; however, B114 displayed markedly greater salt tolerance during subsequent seedling development. Under control conditions, PHT55 exhibited superior basal growth performance compared with B114 (Figure 4C–F), with significantly greater fresh weight (1.9 vs. 1.5 g), dry weight (0.30 vs. 0.23 g), plant height (32.7 vs. 26.3 cm), and root length (27.0 vs. 10.7 cm). Salt stress significantly inhibited growth in both genotypes, but the growth retention rate of B114 was substantially higher than that of PHT55. In B114, plant height decreased from 26.3 to 19.5 cm (25.9% decrease), root length from 10.7 to 9.2 cm (14.0% decrease), and fresh weight from 1.5 to 1.3 g (13.3% decrease), while dry weight remained unchanged at 0.23 g (0% decrease), demonstrating remarkable growth stability under salt stress. In contrast, PHT55 exhibited much greater growth inhibition, with plant height decreasing from 32.7 to 12.6 cm (61.5% decrease), root length from 27.0 to 7.6 cm (71.9% decrease), fresh weight from 1.9 to 0.7 g (63.2% decrease), and dry weight from 0.30 to 0.20 g (33.3% decrease).

### 2.5. Comparative Transcriptome Sequencing of Salt-Tolerant and Salt-Sensitive Maize Inbred Lines

Based on the phenotypic validation results described above, the salt stress responses of the highly salt-tolerant inbred line B114 and the salt-sensitive inbred line PHT55 showed significant differences. To further elucidate the molecular regulatory mechanisms underlying their contrasting salt tolerance, RNA sequencing was performed on samples collected under different treatments and time points, followed by data preprocessing and quality control.

**Figure 5:**
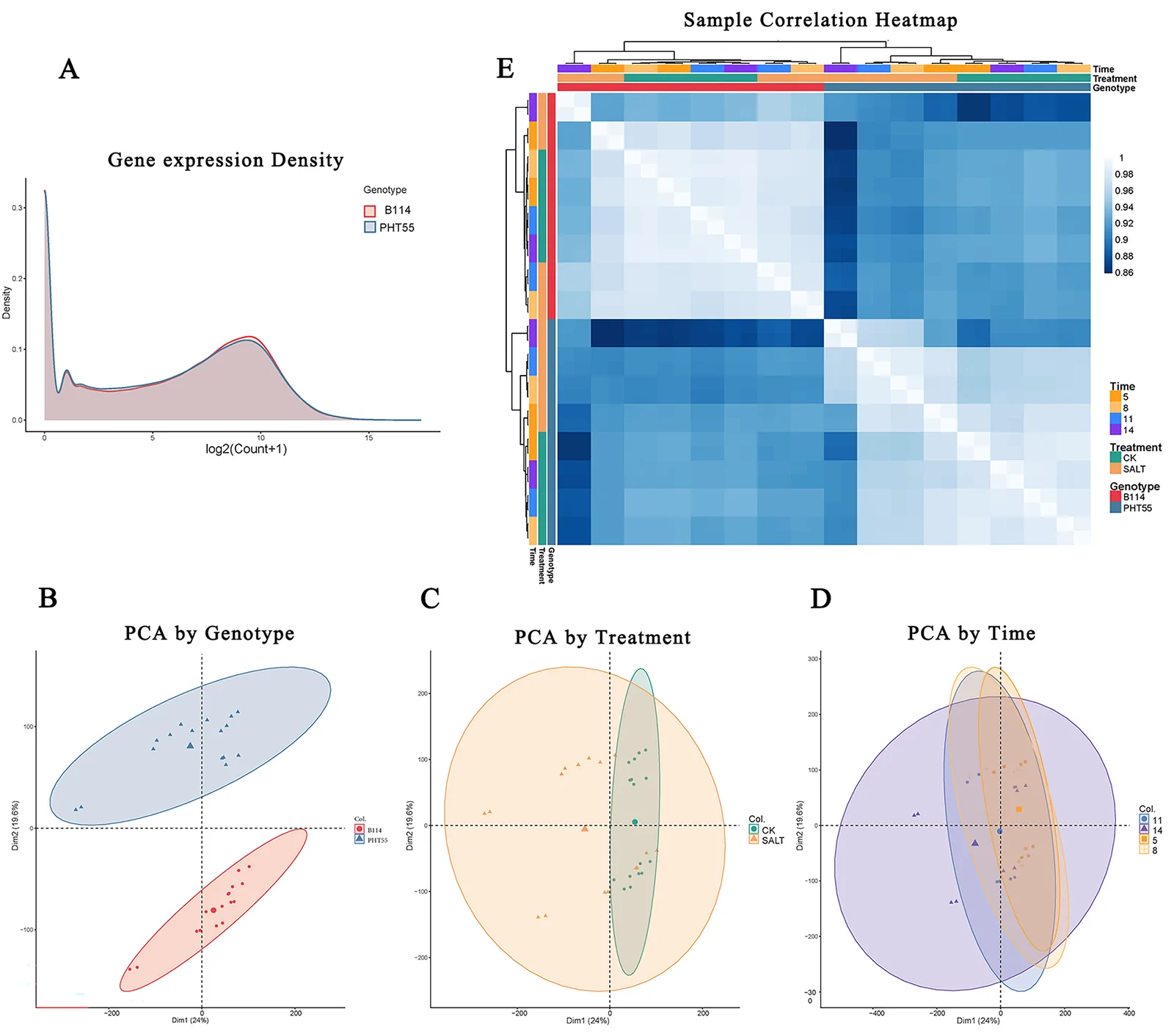
Quality control and exploratory analysis of RNA-seq transcriptome data. (**A**) Density distribution of log_2_-transformed normalized gene expression values (log_2_(Count + 1)) for salt-tolerant inbred line B114 (red) and salt-sensitive inbred line PHT55 (blue). (**B**–**D**) Principal component analysis (PCA) of RNA-seq samples colored by genotype (**B**), treatment (**C**), and sampling time point (**D**). Dim1 and Dim2 represent the first and second principal components, respectively, with the percentage of variance explained indicated in parentheses. (**E**) Sample correlation heatmap based on Pearson correlation coefficients of normalized gene expression values. (2 genotypes × 2 treatments × 4 time points × 2 replicates = 32 samples).

The gene expression density distribution (Figure 5A) showed a typical bimodal pattern in the log2(Count + 1) expression levels across all samples from both inbred lines. The left peak corresponds to lowly expressed genes, while the right peak represents highly expressed genes, consistent with the expected distribution of transcriptomic data. The overall expression density profiles of B114 and PHT55 were highly similar, with no evident shifts or abnormal distributions, indicating stable transcriptome quality across all samples. These results confirm the reliability of the dataset and provide a solid foundation for subsequent comparative analyses between the two genotypes.

The principal component analysis (PCA) results (Figure 5B) showed that the first two principal components, PC1 and PC2, explained 24.0% and 19.6% of the total variance, respectively, with a cumulative contribution of 43.6%, capturing the major differences among samples. When grouped by genotype (Figure 5C), B114 and PHT55 samples formed two completely separated clusters, indicating that genotype is the primary driver of transcriptomic variation and that the two inbred lines exhibit significant differences in gene expression profiles. Grouping by treatment (Figure 5D) revealed clear separation between control (CK) and salt-stressed (Salt) samples, demonstrating that salt stress exerts a strong regulatory effect on gene expression. Furthermore, PCA grouping by time point (Figure 5E) showed discernible clustering trends across different sampling times, reflecting the dynamic nature of gene expression in response to salt stress.

The Pearson correlation heatmap among samples (Figure 5E) showed that all pairwise correlation coefficients were above 0.86, indicating a high overall level of reproducibility and no obvious outlier samples. Moreover, samples from the same genotype and treatment clustered together, with within-group correlations significantly higher than between-group correlations. The fact that samples clustered according to biological conditions rather than sequencing batch, combined with the high within-group correlation coefficients, indicates that technical variation—if present—is substantially smaller than biological variation. This confirms that genotype and treatment are the primary factors driving sample clustering. These results further validate the reliability of the RNA-seq data and support subsequent identification of differentially expressed genes and analyses of salt tolerance regulatory mechanisms.

### 2.6. Differential Gene Expression Analysis of Salt-Tolerant and Salt-Sensitive Maize Inbred Lines Under Salt Stress

To elucidate the transcriptional response differences between the salt-tolerant inbred line B114 and the salt-sensitive inbred line PHT55 under salt stress, differentially expressed genes (DEGs) were identified from transcriptome data at 5, 8, 11, and 14 days of salt treatment (Supplementary Table S5). Volcano plots were used to visualize the overall distribution and quantity of DEGs at each time point.

**Figure 6:**
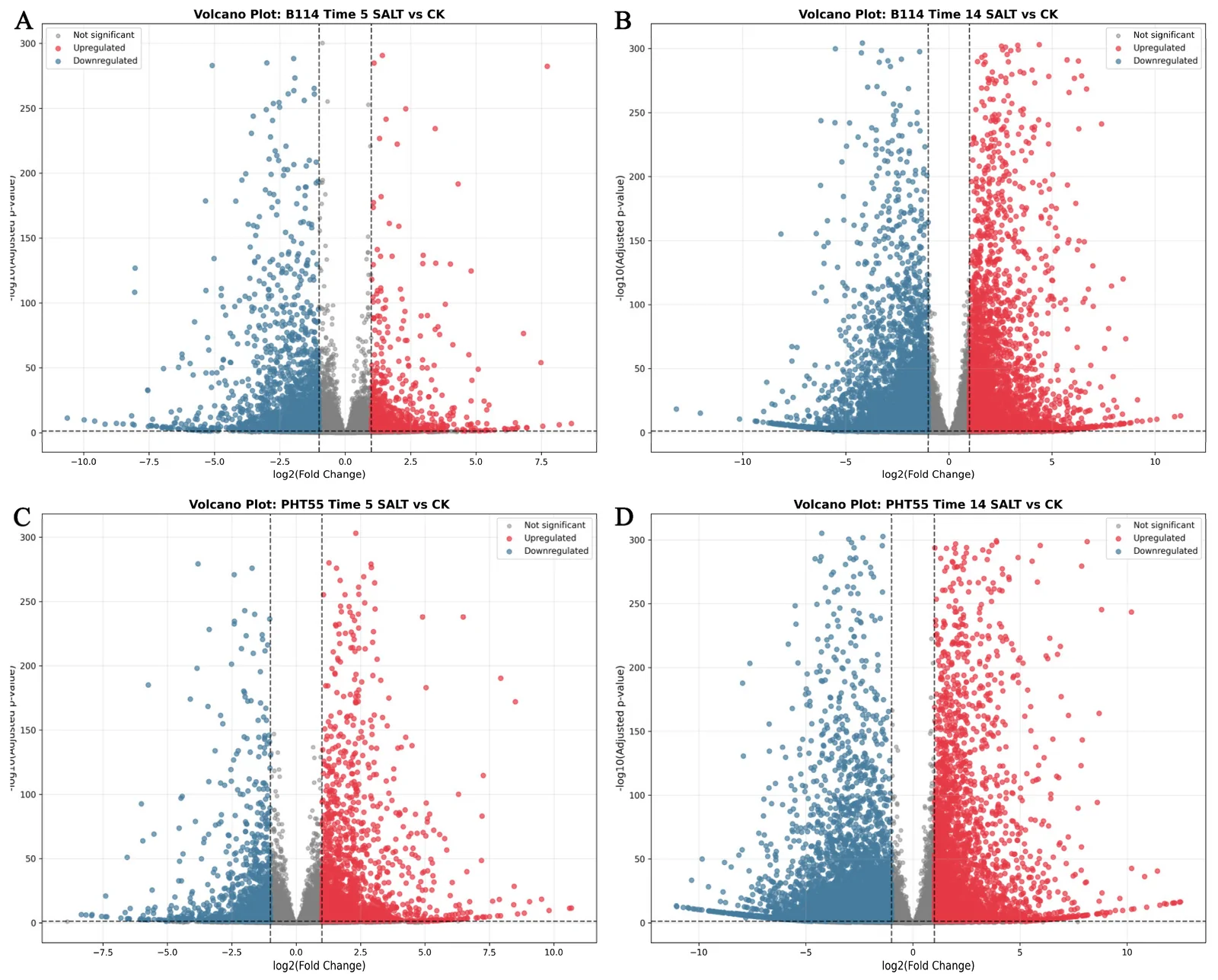
Transcriptomic response differences between salt-tolerant inbred line B114 and salt-sensitive inbred line PHT55 under salt stress. Volcano plots showing differentially expressed genes (DEGs) between salt stress (SALT) and control (CK) conditions. (**A**) B114 at 5 days of salt stress. (**B**) B114 at 14 days of salt stress. (**C**) PHT55 at 5 days of salt stress. (**D**) PHT55 at 14 days of salt stress. The *x*-axis represents log_2_ fold change (log_2_FC); the *y*-axis represents −log_10_ adjusted *p*-value. Vertical dashed lines indicate the fold change threshold (|log_2_FC| > 1); the horizontal dashed line indicates the significance threshold (adjusted *p*-value < 0.05, Benjamini–Hochberg correction). Red points: significantly upregulated genes; blue points: significantly downregulated genes; gray points: non-significant genes. DEGs were identified using DESeq2. Volcano plots for intermediate time points (8 and 11 days) are provided in Supplementary Figure S2.

The results indicated that B114 exhibited a gradual and orderly transcriptional response under salt stress, with relatively low overall transcriptome perturbation and clear temporal dynamics. At day 5 of stress (Figure 6A), a total of 2690 DEGs were detected, including 868 upregulated and 1822 downregulated genes, with downregulated genes predominating, suggesting that at the initial sampling point of the adaptive phase, B114 primarily responds by repressing certain genes to conserve energy. By day 8 (Supplementary Figure S2A), the number of DEGs slightly increased to 2907, with 1591 upregulated and 1316 downregulated genes, indicating the initiation of active upregulation of salt tolerance-related genes. At day 11 (Supplementary Figure S2B), DEGs decreased to 2261, including 1565 upregulated and 696 downregulated genes, reflecting a gradual stabilization of transcriptional regulation. By day 14 (Figure 6B), the number of DEGs surged to 9124, with 4087 upregulated and 5037 downregulated genes, representing a comprehensive transcriptional response, likely reflecting large-scale gene expression reprogramming to adapt to prolonged salt stress.

In contrast to B114, the salt-sensitive inbred line PHT55 exhibited severe transcriptional perturbation and sustained imbalance under salt stress, with a markedly higher degree of transcriptomic disorder throughout the treatment period. At day 5 of stress (Figure 6C), a total of 3728 DEGs were identified, including 2170 upregulated and 1558 downregulated genes, indicating an immediate large-scale transcriptional response and a strong signature of passive stress response. By day 8 (Supplementary Figure S2C), the number of DEGs increased sharply to 6515, with 2435 upregulated and 4080 downregulated genes, suggesting a pronounced disruption of transcriptional homeostasis, particularly reflected by a dramatic increase in downregulated genes. At day 11 (Supplementary Figure S2D), the number of DEGs remained at a high level (5992), including 2410 upregulated and 3582 downregulated genes, indicating sustained high-intensity transcriptional disturbance. By day 14 (Figure 6D), DEGs reached a peak of 10,108, comprising 4196 upregulated and 5912 downregulated genes, substantially exceeding those observed in B114 at the same stage, further suggesting exacerbated transcriptional dysregulation under prolonged salt stress.

**Figure 7:**
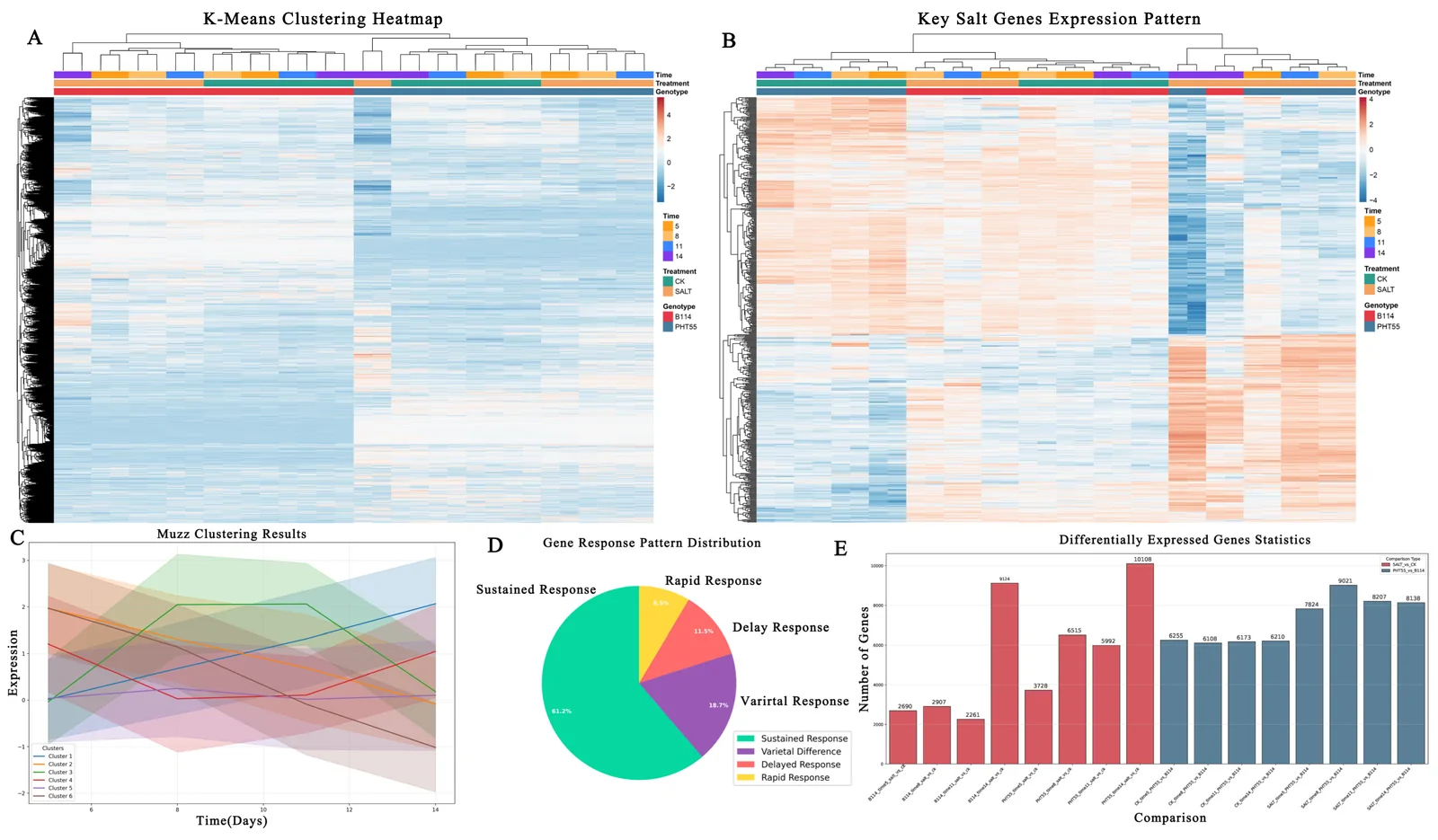
Dynamic transcriptome clustering analysis of salt-tolerant B114 and salt-sensitive PHT55 maize inbred lines under salt stress. (**A**) K-means clustering heatmap of all differentially expressed genes (DEGs) across all samples. The color scale represents Z-score normalized expression values (blue: low; red: high). The top annotation bars indicate time point (5, 8, 11, 14 days), treatment (CK, control; SALT, salt stress), and genotype (B114, PHT55). (**B**) Expression heatmap of key salt stress-responsive genes, displaying distinct genotype- and time-specific expression patterns under control and salt stress conditions. (**C**) Mfuzz soft clustering of DEG expression trajectories over the 14-day salt stress time course, showing nine distinct temporal clusters with centroid lines (solid) and standard deviation regions (shaded). (**D**) Pie chart showing the proportional distribution of four gene response pattern categories: Sustained Response, Varietal Response, Delayed Response, and Rapid Response. (**E**) Bar plot showing the number of upregulated (red) and downregulated (blue) DEGs across all comparison groups (B114 and PHT55 at 5, 8, 11, and 14 days of salt stress vs. CK). Volcano plots comparing PHT55 and B114 genotypes under control and salt stress conditions are provided in Supplementary Figure S3.

To further validate the overall reliability of the transcriptome data, we conducted sample correlation analysis and principal component analysis (PCA). The sample correlation heatmap (Supplementary Figure S2E) showed tight clustering of replicates within the same genotype and treatment, indicating high consistency among biological replicates. PCA (Supplementary Figure S2F) further demonstrated clear separation between different genotypes and treatments, with a pronounced temporal gradient effect, indicating that experimental treatment had a stronger impact on the transcriptome than batch effects and confirming the high quality of the data. Hierarchical clustering heatmaps of DEGs (Figure 7A) revealed distinct global expression patterns between the two inbred lines across time points: the highly salt-tolerant line B114 exhibited relatively stable and moderate transcriptional changes, whereas the salt-sensitive line PHT55 displayed more drastic and widespread fluctuations. Analysis of key salt tolerance-related genes (Figure 7B) further confirmed that B114 maintained an orderly and sustained regulatory pattern, while PHT55 showed highly variable and imbalanced transcriptional responses under salt stress.

A comparative analysis revealed that the two inbred lines exhibit fundamentally distinct transcriptional response patterns under salt stress. During the early stress phase (5–8 days), PHT55 displayed a substantially higher number of DEGs than B114, reflecting a rapid, intense, and largely passive stress response. In the later phase of salt stress (11–14 days), both lines initiated large-scale transcriptional reprogramming; however, B114 maintained transcriptional homeostasis through a balanced ratio of up- and down-regulated genes, whereas PHT55 was dominated by down-regulated genes, indicating severe transcriptional imbalance. These transcriptional patterns align closely with the observed phenotypic and physiological data, confirming that orderly and moderate transcriptional regulation underpins the high salt tolerance of B114, while transcriptional dysregulation contributes to the salt sensitivity of PHT55.

### 2.7. Temporal Expression Patterns of Differentially Expressed Genes Under Salt Stress

Based on the identification of differentially expressed genes (DEGs) at the aforementioned time points, we further analyzed the dynamic transcriptional regulatory responses of the highly salt-tolerant inbred line B114 and the salt-sensitive inbred line PHT55 under salt stress. The DEG expression data from both lines at 5, 8, 11, and 14 days of salt treatment were integrated to construct a temporal expression matrix, which was subsequently subjected to time-series expression pattern clustering and response characteristic analysis, as detailed in Supplementary Table S6.

Volcano plots were used to visualize the overall transcriptional differences between the two inbred lines under different treatments and time points (Supplementary Figure S3A–H), and the dynamics of DEG numbers were statistically summarized (Supplementary Figure S3E). Subsequently, K-means clustering was performed on the integrated DEG dataset (Figure 7A), and a separate clustering analysis was conducted for key salt stress-responsive genes (Figure 7B). The results showed that all DEGs were classified into multiple distinct expression modules based on their temporal expression patterns. Genes within the same module exhibited highly consistent expression trajectories, whereas clear differences were observed among modules. In B114, most DEGs displayed a pattern of low early expression followed by orderly upregulation at later stages, with relatively smooth expression dynamics and no drastic fluctuations, indicating a well-regulated transcriptional response. In contrast, PHT55 exhibited a pattern characterized by strong early fluctuations and sustained transcriptional disorganization at later stages. In several modules, genes were sharply upregulated at early stages of stress and subsequently downregulated, reflecting severe disruption of transcriptional homeostasis.

To further dissect the dynamic response patterns of DEGs, temporal expression data were subjected to Mfuzz clustering analysis (Figure 7D), and gene response patterns were classified and quantified based on the clustering results (Figure 7C). The analysis revealed that key genes in different modules exhibited distinct response trends: some genes were significantly upregulated at the early adaptive phase and maintained high expression throughout the stress period, suggesting their involvement in early salt stress signal perception and transduction. Other genes showed sustained high expression only at later stages, likely participating in adaptation to prolonged stress. Additionally, some genes were consistently downregulated under salt stress, potentially contributing to growth inhibition under adverse conditions. Importantly, the same genes often displayed markedly different expression patterns between the two genotypes. For example, certain salt-tolerance-related genes that were upregulated over time in B114 were continuously downregulated in PHT55, directly reflecting the fundamental differences in transcriptional regulatory strategies employed by the two genotypes under salt stress.

### 2.8. Expression Pattern Analysis of DEGs Under Salt Stress

To further elucidate the temporal expression patterns of differentially expressed genes (DEGs) in the salt-tolerant inbred line B114 and the salt-sensitive inbred line PHT55 and to identify key genes underlying differences in salt tolerance, a comprehensive analysis was performed based on the previously identified DEGs, including expression normalization, temporal trend clustering, expression pattern characterization, and highly variable gene screening. To minimize the influence of sequencing depth variation and batch effects, raw gene expression data from all samples were normalized, generating a standardized expression matrix (Supplementary Table S7).

Based on the normalized expression matrix, two complementary clustering approaches, Mfuzz clustering (Figure 7D) and K-means clustering (Figure 7A), were employed to classify DEGs according to their temporal expression trajectories under salt stress, thereby characterizing their dynamic transcriptional responses (Supplementary Table S8). Comparative analysis revealed a high degree of concordance between the two clustering methods. Most genes were consistently assigned to the same expression modules by both approaches, whereas only a small subset showed differences in module assignment.

We quantified the number of genes and their distribution within each expression module based on the clustering results, thereby defining distinct types of salt stress responses (Supplementary Table S8). The results (Figure 7E) show that gene numbers vary across different modules, with each module representing a characteristic temporal response and regulatory pattern under salt stress, clearly distinguishing the timing and regulatory features of gene responses. Using the coefficient of variation as an indicator, we evaluated the expression fluctuation of genes across different samples and time points, enabling the identification of highly variable genes (Supplementary Table S9). By integrating the clustering results with expression variability, some key salt stress-responsive genes were selected from the pool of all DEGs, and their differential expression patterns were visualized through a heatmap (Figure 7B).

Furthermore, GO enrichment analysis was performed on the DEGs identified in each comparison group to characterize the functional pathways associated with salt stress responses in both genotypes (Supplementary Figure S4). In B114, oxidative stress and redox metabolism-related terms dominated the enrichment profiles across all time points, with “iron ion binding,” “monooxygenase activity,” and “oxidoreductase activity” consistently ranking as the top three terms (GeneRatio: 0.038–0.042 at day 5, gradually decreasing to 0.015–0.020 by day 14). Notably, “response to oxidative stress,” “cellular oxidant detoxification,” “peroxidase activity,” and “hydrogen peroxide catabolic process” were enriched throughout the 14-day treatment, indicating a sustained enzymatic antioxidant defense. Additionally, “regulation of jasmonic acid-mediated signaling pathway” was specifically enriched at day 5 in B114 but absent in PHT55. In contrast, PHT55 exhibited fewer oxidative stress-related terms (4–5 of 10 enriched terms versus 7–8 in B114) and showed unique enrichment in “quercetin 7-O-glucosyltransferase activity” and “UDP-glycosyltransferase activity,” suggesting a greater reliance on flavonoid-based secondary metabolite scavenging. The genotype comparison (PHT55 vs. B114 under salt stress) revealed that “iron ion binding” was the top-ranked enriched term at all four time points, while “water channel activity” and “water transport” were specifically enriched at day 11 and “protein autophosphorylation” at day 5, highlighting key functional divergences between the two genotypes in iron homeostasis, water transport regulation, and kinase-mediated signaling under salt stress.

### 2.9. Random Forest-Based Screening of Key Salt-Tolerance Genes

In conventional transcriptomic studies, traditional differential expression and functional enrichment analyses can identify a subset of candidate genes; however, these approaches have inherent limitations. Specifically, they rely heavily on user-defined thresholds, such as fold-change values and adjusted *p*-value cutoffs, which may lead to the exclusion of genes with subtle expression changes but critical regulatory functions. Moreover, these methods are often inadequate for systematically and unbiasedly identifying the core regulatory genes underlying genotypic differences in salt tolerance from high-dimensional transcriptomic datasets.

Building on the previous results from expression pattern clustering and functional enrichment analyses, and aiming to overcome the limitations of conventional gene selection methods, we further aimed to identify key genes mediating the salt tolerance differences between the highly tolerant inbred line B114 and the sensitive inbred line PHT55 from high-dimensional transcriptomic data. To achieve this, we applied the Random Forest machine learning algorithm, using the standardized expression matrix of 16,194 significantly differentially expressed genes (DEGs, |log_2_FC| ≥ 1, adjusted *p* < 0.05) as input features. A treatment classification model (SALT vs. CK) was constructed, and feature importance analysis was performed to systematically and unbiasedly screen for core salt stress-responsive genes.

**Figure 8:**
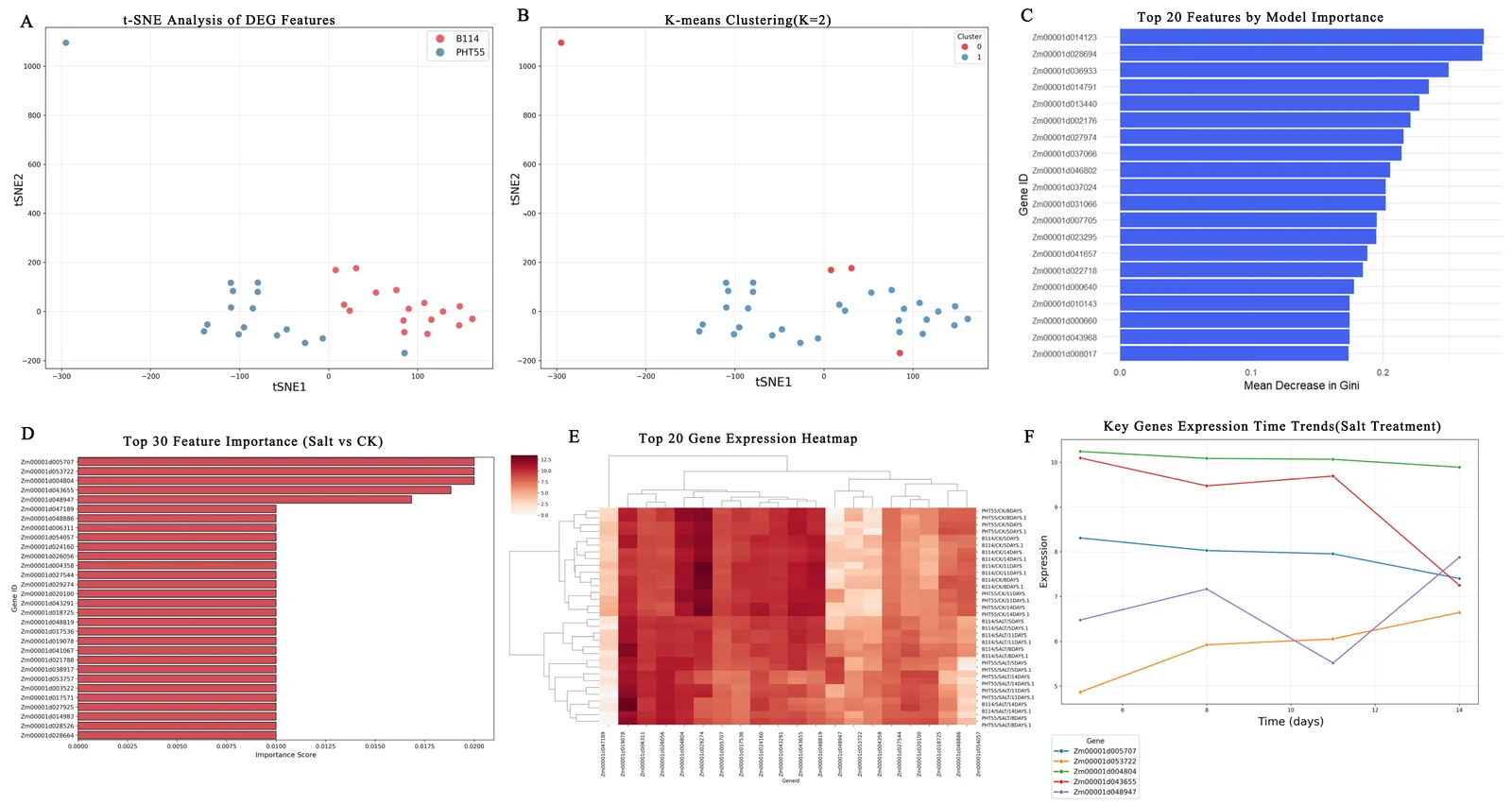
Machine learning-based screening and validation of core salt tolerance genes in maize. (**A**) t-SNE dimensionality reduction visualization of DEG features, showing sample separation between salt-tolerant inbred line B114 (red) and salt-sensitive inbred line PHT55 (blue). (**B**) K-means clustering results (K = 2) based on gene expression features, confirming genotype-based grouping. (**C**) Top 20 core feature genes ranked by Random Forest mean decrease in Gini importance. (**D**) Top 30 genes ranked by feature importance score under salt stress (Salt vs. CK comparison). (**E**) Hierarchical clustering heatmap of the top 20 core salt tolerance-related genes across all samples, with color scale representing normalized expression levels (red: high; white: low). (**F**) Time-course expression profiles of five key salt stress-responsive genes under salt stress treatment (5, 8, 11, and 14 days), with distinct line colors and markers representing individual genes. Gene IDs are prefixed with “Zm”, denoting Zea mays gene identifiers. DEG = differentially expressed gene; t-SNE = t-distributed stochastic neighbor embedding; CK = control.

A machine learning feature matrix was constructed based on the standardized expression levels of 16,194 DEGs across 32 samples (Supplementary Table S10). To ensure the validity of the modeling data, we first performed sample grouping and clustering validation. t-SNE dimensionality reduction analysis was used to visualize the distribution of sample features (Figure 8A), showing clear separation between the highly salt-tolerant inbred line B114 (red) and the salt-sensitive inbred line PHT55 (blue), with no obvious overlap. This indicates that the feature matrix effectively captures genotype-specific transcriptional signatures, meeting the requirements for downstream modeling. Additionally, K-means clustering based on the feature matrix (Figure 8B) divided the samples into two modules (K = 2), with highly consistent expression trends within each module, further confirming the reliability of genotype-based grouping.

Based on the constructed feature matrix, we performed Random Forest model training and feature importance ranking. The dataset was split into training (70%) and testing (30%) sets, and 5-fold cross-validation was applied to assess model stability. The Random Forest classifier (n_estimators = 100, random_state = 42) was trained to distinguish between salt-stressed and control treatments. Feature importance scores were derived from the Gini impurity criterion, and the top 20 genes were visualized (Figure 8C), highlighting the key genes contributing most to treatment differentiation. Under salt stress, the top 30 genes ranked by feature importance were further selected to identify key genes directly associated with the salt stress response (Figure 8D, Supplementary Table S11).

The expression characteristics of the identified key salt-tolerance genes were validated from multiple dimensions. The heatmap displays the abundance of gene expression across different genotypes, treatments, and time points (Figure 8E). The results show significant expression differences between B114 and PHT55, with clear temporal gradients under salt stress, closely correlating with salt-tolerant phenotypes. The temporal expression patterns of key salt-responsive genes under salt stress revealed distinct response modes, including sustained upregulation, early response, and late induction (Figure 8F). The combined validation of expression patterns and temporal dynamics indicates that these genes are strongly associated with salt stress responses and provide precise candidate targets for elucidating the molecular mechanisms of maize salt tolerance.

### 2.10. Expression Validation of Key Salt-Tolerance Genes by qRT-PCR

To validate the reliability of the RNA-seq results and to further characterize the dynamic response patterns of key salt-tolerance genes in the maize reference inbred line B73, ten representative genes were selected from the salt-tolerance gene set identified by the Random Forest algorithm. Priority was given to genes with annotated functions closely associated with salt stress responses, including ion transport, redox homeostasis, and stress signal transduction, as well as those exhibiting representative expression patterns. These genes were subsequently subjected to qRT-PCR validation.

**Figure 9:**
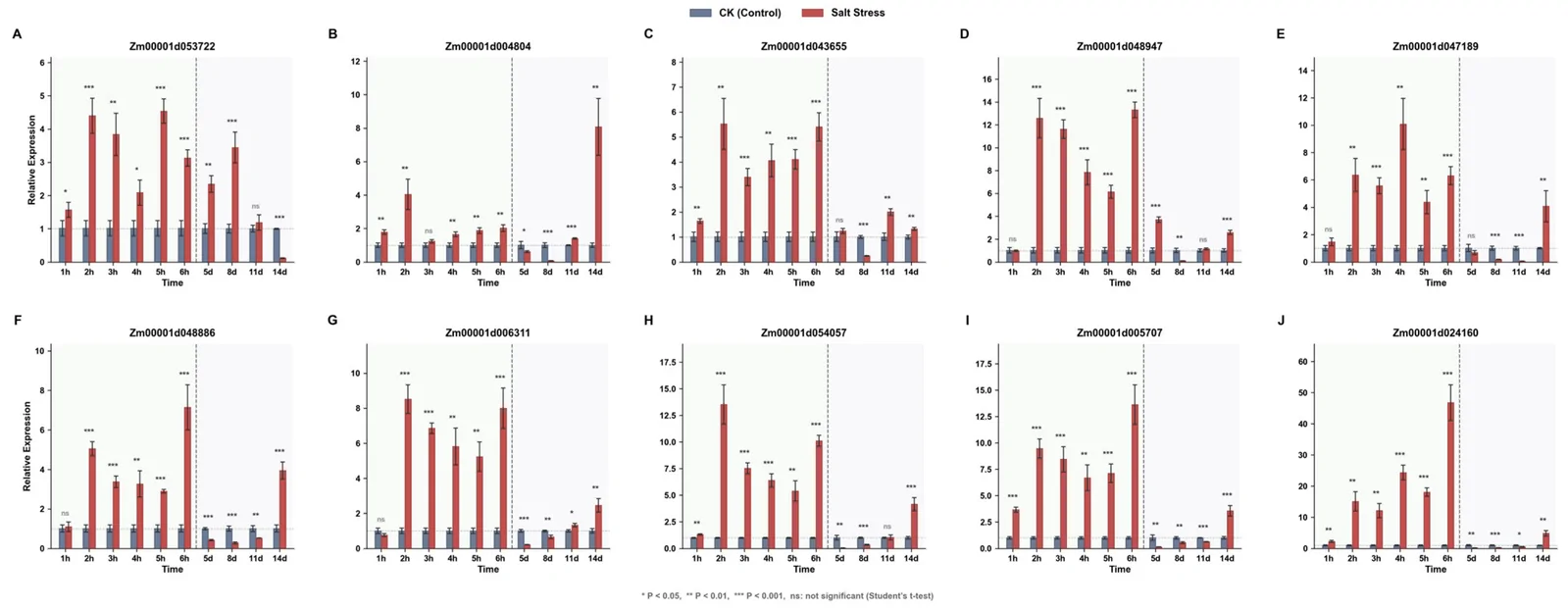
qRT-PCR validation of 10 candidate salt tolerance genes in maize under salt stress. (**A**–**J**) Relative expression of genes ML1–ML10 measured by qRT-PCR. Bars show the mean ± SD of three biological replicates. Blue-gray: CK (control); red: Salt stress. The *X*-axis includes short-term (1–6 h) and long-term (5–14 d) treatments, separated by the vertical dashed line. Expression was calculated using the 2^−ΔΔCt^ method with Actin as the reference gene. Short-term data were normalized to a single CK; long-term data were normalized to time-matched CK. Significance was determined by Student’s *t*-test (* *p* < 0.05, ** *p* < 0.01, *** *p* < 0.001, ns: not significant). Each panel uses an independent *y*-axis scale. Gene IDs: (**A**) Zm00001d053722, (**B**) Zm00001d004804, (**C**) Zm00001d043655, (**D**) Zm00001d048947, (**E**) Zm00001d047189, (**F**) Zm00001d048886, (**G**) Zm00001d006311, (**H**) Zm00001d054057, (**I**) Zm00001d005707, (**J**) Zm00001d024160.

The qRT-PCR results confirmed that all ten candidate genes exhibited clear salt stress-responsive patterns in maize (Figure 9A–J, Supplementary Table S12). During the short-term phase (1–6 h), most genes showed rapid and significant induction, with peak fold-changes ranging from approximately 4- to 48-fold. Zm00001d024160 exhibited the strongest induction, peaking at 5 h (48-fold), followed by Zm00001d054057 and Zm00001d005707 (both 14-fold at 2 h and 5 h, respectively). Zm00001d048947 also displayed robust early induction (13-fold at 2 h and 5 h). During the long-term phase (5–14 d), expression of most genes was attenuated, with several genes returning to near-baseline levels at days 5–11. Notably, a subset of genes showed significant late reactivation at day 14, including Zm00001d004804 (8-fold), Zm00001d047189 (4-fold), and Zm00001d048886 (4.5-fold). These biphasic temporal profiles—characterized by strong early transcriptional activation followed by mid-term attenuation and partial late recovery—suggest that the candidate genes function at different stages of the salt stress adaptive response, from initial stress signaling to long-term acclimation.

## 3. Discussion

This study established a mixed-salt stress system (NaCl:Na_2_SO_4_ = 9:1) that mirrors the ionic composition of saline groundwater in the Yellow River Delta, providing a field-relevant platform for maize salt tolerance evaluation. Using this system, 143 maize inbred lines were classified into four tolerance levels based on a multi-trait comprehensive salt tolerance index (D value), from which the highly tolerant line B114 and the sensitive line PHT55 were selected as contrasting genotypes for transcriptomic analysis. This multi-trait approach reduces the limitations of single-trait evaluation and better aligns with the complex nature of salt tolerance in field conditions.

Time-series transcriptome analysis revealed distinct temporal expression patterns between B114 and PHT55. B114 exhibited relatively stable transcriptional changes with gradual regulatory progression, whereas PHT55 displayed more pronounced early transcriptional responses and a higher number of DEGs at later time points. These differences in temporal dynamics may reflect divergent stress adaptive strategies between the two genotypes, though the functional significance of these transcriptional differences requires further validation. Compared with conventional single time-point analyses, the time-series approach better captured the dynamic nature of transcriptional responses under prolonged salt stress.

Integration of random forest machine learning with transcriptomic data complemented traditional DEG screening by prioritizing candidate genes that may contribute to genotypic differences in salt tolerance. This approach enabled the identification of 50 candidate genes for further investigation, though functional validation through gene editing or transgenic approaches is needed to confirm their roles in salt tolerance mechanisms. In this study, we integrated the Random Forest machine learning algorithm with time-series transcriptomic data to construct a genotype classification model and identify core salt-tolerance genes based on feature importance analysis, thereby overcoming these limitations. The results demonstrated that the Random Forest model could effectively discriminate the transcriptional profiles of B114 and PHT55, and cross-validation confirmed its robust performance. Notably, the top 50 genes ranked by feature importance were highly consistent with previous expression pattern clustering and GO enrichment analyses, with most genes enriched in salt stress-related pathways, including redox regulation, ion homeostasis maintenance, and stress signal transduction, thereby validating the biological relevance of the selected gene set. This approach enables unbiased and objective identification of key regulatory genes from high-dimensional datasets, providing a new strategy for transcriptomic studies of plant stress responses and offering precise candidate targets for subsequent functional validation of maize salt-tolerance genes.

However, this study also has certain limitations. First, salt tolerance evaluation was conducted only at the seedling stage, which may not fully represent whole-life-cycle salt tolerance in the field; subsequent studies should combine field trials to validate adult-plant performance [10,37]. Second, the identified key salt-tolerance genes were validated only through expression patterns and functional enrichment analyses, without functional verification such as gene editing or overexpression, so their precise regulatory mechanisms in maize salt tolerance remain to be elucidated [17,52,53]. Third, the mixed-salt stress system was specifically designed to match the ionic composition of the Dongying saline-alkali soils, and its applicability to other regions with different soil salt compositions needs further testing [11,12]. Finally, the machine learning model was built on transcriptome data from this study, and its generalizability across different genetic backgrounds and stress conditions remains to be assessed [54].

Notably, root tissues were selected for transcriptomic analysis because the root serves as the primary site of Na^+^ uptake and exclusion, xylem loading, and rhizosphere salt perception—processes that are central to ion-homeostasis maintenance under salt stress. The transcriptomic data from root tissues thus provide direct insights into the molecular regulatory networks governing these root-mediated salt responses. The identification of core genes enriched in ion homeostasis and ion transport pathways further validates the suitability of root-based transcriptomics for dissecting the mechanisms of salt tolerance in maize.

Future research can focus on the following directions: breeding applications using core salt-tolerant parents; utilizing highly salt-tolerant germplasm to develop new maize varieties adapted to saline-alkali soils and integrating molecular marker-assisted selection for efficient breeding [18,19,51]; functional validation of key salt-tolerance genes; conducting gene functional studies to elucidate regulatory pathways, providing precise targets for molecularly guided maize salt-tolerance improvement [17,55,56]; expansion and optimization of the stress evaluation system; adapting the salt-stress evaluation system to diverse saline-alkali soils by considering regional ionic compositions and establishing a multi-regional maize salt-tolerance screening platform [9,10,11]; seedling-to-adult-stage correlation modeling; and integrating field trial data to build models linking seedling-stage and adult-stage salt tolerance, thereby improving the practical utility of the evaluation system [37,57].

## 4. Materials and Methods

### 4.1. Plant Materials and Salt Stress Treatment

Salt Solution System Design: Based on extensive literature surveys, preliminary dose–response experiments, and compositional analyses of groundwater from the Yellow River Delta region, a mixed-salt stress system was designed to provide standardized, reproducible, and regionally relevant experimental conditions. Groundwater samples were collected from Dongying, Shandong Province, and electrical conductivity, resistivity, total dissolved solids, salinity, and pH were measured using a calibrated portable multiparameter water quality meter [4]. The same parameters were determined for single-salt solutions, including 150 mM NaCl and 150 mM Na_2_CO_3_. Prior to large-scale screening, preliminary experiments were conducted using local groundwater diluted to different electrical conductivity levels (EC = 12.65, 14.89, 19.01, and 27.44 dS/m; Supplementary Table S1) to determine the optimal screening concentration. Based on these analyses, a 150 mM mixed-salt solution (NaCl:Na_2_SO_4_ = 9:1, EC = 16.78 dS/m, pH = 7.43) was selected for all subsequent experiments, as it best represented the typical mid-to-high salinity conditions of coastal saline-alkali soils in the Yellow River Delta while providing sufficient stress intensity to differentiate tolerance levels among genotypes without causing complete lethality.

Material Preparation and Grouping: Plastic pots measuring 10 cm × 10 cm × 12 cm (length × width × height) were used, each filled to three-quarters of their volume with vermiculite (particle size: 2–4 mm). Two experimental groups were established—a control group and a treatment group—to create contrasting growth environments. The control group was irrigated with 150 mL of deionized water per pot, while the treatment group received 150 mL of a 150 mM mixed-salt solution (EC = 16.78 dS/m, pH = 7.43) per pot, enabling the assessment of salt tolerance performance among maize germplasm accessions under stress conditions.

Seedling Transplantation: Seedlings germinated for 3 days with uniform growth vigor were selected as screening materials to ensure a consistent initial state across all samples. During transplantation, seedlings were placed into the pre-prepared pots with the radicle oriented downward at a density of 4 seedlings per pot. Pots were subsequently top-filled with vermiculite (particle size: 2–4 mm) to the level of the rim, providing a suitable substrate for experimental plant growth.

Seedling-Stage Cultivation Management: All pots containing transplanted seedlings were transferred to a controlled-environment growth chamber with precisely regulated conditions. The chamber temperature was maintained at a constant 27.3 °C, with relative humidity held at 54.5%. Each shelf of the cultivation rack was equipped with five supplemental light fixtures, delivering a measured light intensity of ≥12,500 lux (approximately 208 μmol·m^−2^·s^−1^ of photosynthetic photon flux density) at the maize seedling canopy level. The photoperiod was set to 16 h light/8 h dark. Throughout the cultivation period, 100 mL of deionized water was supplemented to each control-group pot every 5 days to ensure adequate water supply and normal growth, while 100 mL of a 150 mM mixed-salt solution (EC = 16.78 dS/m, pH = 7.43) was supplemented to each treatment-group pot on the same schedule to sustain continuous salt stress. The entire treatment lasted 14 days, during which phenotypic observations of all germplasm accessions were conducted and recorded.

### 4.2. Measurement of Salt-Tolerance-Related Growth Traits

Six growth-related traits were measured to evaluate salt tolerance: germination vigor (3 days), germination rate (7 days), plant height (14 days), primary root length (14 days), fresh weight (14 days), and dry weight (14 days). For the germination assay, 50 seeds per accession were germinated with either deionized water (control) or a 150 mM NaCl solution (EC = 14.69 dS/m, pH = 6.98), and the number of germinated seeds was recorded daily. Plant height and root length were measured using a ruler. Fresh weight was determined immediately after sample collection. Following fresh weight measurement, whole plants were placed in kraft paper bags, subjected to an initial fixation step in a forced-air oven at 120 °C for 10 min, then transferred to an oven set at 80 °C and dried to constant weight. Dry weight was subsequently measured using an electronic balance with a precision of 0.001 g. For all phenotypic trait measurements, more than 10 biological replicates were established, with each biological replicate consisting of 3 pots (12 seedlings total per replicate). From each biological replicate, a minimum of 5 seedlings were randomly selected for trait determination, and mean values were calculated thereafter.

The comprehensive salt tolerance index (D value) was calculated using the membership function method [38,41]. First, relative values for each trait were calculated as the ratio of salt-stress to control measurements (Salt/CK). Three membership function values were then computed: μ_1_ (plant height), μ_2_ (root length), and μ_3_ (biomass, integrating fresh weight, dry weight, and fresh-to-dry weight ratio). For each trait, the membership function value was calculated as: μ(X) = (X − Xmin)/(Xmax − Xmin), where X is the relative value of a given trait for a specific inbred line, and Xmax and Xmin are the maximum and minimum values of that trait across all 143 inbred lines, respectively. The D value was then calculated as the arithmetic mean of the three membership values: D = (μ_1_ + μ_2_ + μ_3_)/3. The D value ranges from 0 to 1, with higher values indicating stronger comprehensive salt tolerance.

### 4.3. RNA Sequencing and Data Analysis

Root samples were collected from two maize inbred lines (B114 and PHT55) under control (CK) and salt stress (Salt) conditions at four time points (5, 8, 11, and 14 days after treatment), with two biological replicates per condition, yielding a total of 32 samples. The raw sequencing data have been deposited in the NCBI Sequence Read Archive (SRA) under BioProject accession number PRJNA1508389. Total RNA was extracted from root samples using TRIzol reagent (Thermo Fisher Scientific, Waltham, MA, USA) according to the manufacturer’s instructions. RNA integrity was assessed using an Agilent 2100 Bioanalyzer (Santa Clara, CA, USA), and samples with RNA integrity number (RIN) ≥ 7.0 were retained for library construction. Sequencing libraries were prepared using the NEBNext Ultra RNA Library Prep Kit for Illumina (NEB, USA) and sequenced on the Illumina NovaSeq 6000 platform to generate 150 bp paired-end reads.

For data processing, raw sequencing reads were first evaluated for quality using FastQC (version 0.11.9). Adapter sequences and low-quality bases were trimmed using fastp (version 0.23.0) [58] with default parameters. The cleaned reads were aligned to the maize B73 reference genome (RefGen_v5, Zm-B73-REFERENCE-NAM-5.0) using HISAT2 (version 2.2.1) [59] with default parameters. Gene-level read counts were quantified using featureCounts (version 2.0.3) [60] based on the corresponding gene annotation file. Expression normalization and downstream differential expression analysis were performed using DESeq2 (version 1.38.0) [61] in R. Genes with low expression (total count < 10 across all samples) were filtered out prior to normalization.

### 4.4. Time-Series Transcriptome Analysis

To capture the dynamic changes in gene expression during the salt stress response, a time-series transcriptome analysis was conducted. Expression data from all time points (5, 8, 11, and 14 days) and treatments (CK and SALT) were normalized. Genes with a coefficient of variation (CV) > 0.5 across samples were retained for further analysis. Time-series clustering was performed using K-means clustering and the Mfuzz algorithm to identify gene groups exhibiting similar expression patterns. The analysis was carried out across three dimensions: (1) single-time-point differential expression analysis, comparing the differences between salt stress and control at each time point; (2) temporal dynamic analysis, identifying genes that changed significantly over time; and (3) inter-accession differential analysis, comparing expression differences between B114 and PHT55 at each time point.

### 4.5. Quantitative Real-Time PCR Validation

To validate the RNA-seq results, qRT-PCR analysis was performed on 10 candidate genes using maize B73 as the experimental material. Total RNA was extracted using TRIzol reagent, and reverse transcription into cDNA was carried out using SPARKscript II All-in-One RT SuperMix for qPCR (with gDNA Eraser; SparkJade, Shandong Sikejie Biotechnology Co., Ltd., Jinan, China). qRT-PCR reactions were conducted on the Applied Biosystems^®^ QuantStudio™ 6 Flex system using ChamQ Universal SYBR qPCR Master Mix. The maize β-Actin gene (Zm00001eb220480) was used as the internal reference gene for normalization. Relative expression levels were calculated using the 2^−ΔΔCt^ method. Three technical replicates were performed for each sample.

### 4.6. Statistical Analysis

Statistical analyses were performed using R software (version 4.2.1) and Python (version 3.9). Data are presented as mean ± standard deviation (SD). Pairwise comparisons were conducted using Student’s *t*-test, and multiple comparisons were performed using analysis of variance (ANOVA). Correlation analysis was carried out using Pearson’s correlation coefficient. Principal component analysis (PCA) was employed to visualize the relationships among samples. All figures and plots were generated using the ggplot2 [62] package in R and the matplotlib library [63] in Python.

B73 was chosen for validation because it is the reference genotype for the genome annotation used in this study (RefGen_v5), ensuring exact primer–template matching, and its use as a third genotype independent of the RNA-seq comparison demonstrates that the candidate genes’ salt responsiveness is not restricted to B114 or PHT55.

### 4.7. Machine Learning-Based Screening of Key Salt-Tolerance Genes

A Random Forest classifier was implemented using the scikit-learn library (version 1.1.3) in Python (version 3.9) to identify key genes contributing to salt stress responses. The feature matrix was constructed using the standardized expression levels (Z-score normalized) of 16,194 significantly differentially expressed genes (DEGs; |log_2_FC| ≥ 1, adjusted *p* < 0.05) across all 32 samples as input features, with treatment group (SALT vs. CK) as the classification label. Prior to model training, t-SNE dimensionality reduction and K-means clustering (K = 2) were performed to verify the separability of sample groups in the feature space.

The dataset was randomly split into training (70%) and testing (30%) sets, with 5-fold cross-validation applied on the training set to evaluate model stability. The Random Forest classifier was configured with nestimators = 100, criterion = ‘gini’, and random_state = 42. Feature importance scores were derived from the mean decrease in Gini impurity, and the top 30 genes ranked by importance were selected as core salt tolerance-related candidate genes. Model performance was evaluated using classification accuracy on the testing set.

## 5. Conclusions

In this study, we established a mixed-salt stress evaluation system (NaCl:Na_2_SO_4_ = 9:1) that mirrors the ionic composition of groundwater in the Yellow River Delta, providing a field-relevant phenotyping platform for assessing maize seedling salt tolerance [4,14,22]. Using a multi-trait subordinate function combined with a comprehensive salt tolerance index (D value), 143 maize inbred lines were classified into four tolerance grades, among which the highly tolerant line B114 and the sensitive line PHT55 were identified as contrasting genotypes for in-depth analysis. Relative fresh weight and relative plant height emerged as the principal contributors to the D value, highlighting their utility as reliable and accessible indicators for large-scale salt tolerance screening.

Time-series transcriptomics revealed that “ordered temporal transcriptional regulation” constitutes a core molecular hallmark distinguishing tolerant from sensitive genotypes. The tolerant line B114 exhibited a pattern of steady transcriptional initiation and orderly regulatory progression, whereas the sensitive line PHT55 displayed early drastic transcriptional perturbation followed by progressive regulatory imbalance. Integration of transcriptomic data with random forest machine learning enabled the unbiased identification of 50 core salt tolerance genes; GO enrichment analysis further revealed that B114’s superior salt tolerance is attributable to a multi-layered defense strategy, encompassing sustained enzymatic ROS detoxification (including response to oxidative stress, peroxidase activity, and hydrogen peroxide catabolic process), iron homeostasis maintenance through Fenton chemistry regulation, early activation of jasmonic acid-mediated signaling, and differential aquaporin-mediated water transport regulation (Supplementary Figure S4). qRT-PCR validation of 10 representative candidate genes confirmed their salt-responsive expression patterns, supporting the reliability of the combined RNA-seq and machine learning pipeline.

Collectively, this work provides elite salt-tolerant germplasm resources and a prioritized set of candidate genes underpinning salt tolerance in maize, laying a theoretical and genetic foundation for molecular breeding of salt-alkali-tolerant maize varieties [5,18,19,51]. Future studies incorporating functional validation of the identified genes (e.g., via gene editing or overexpression) and multi-environment field trials will further accelerate their application in saline-alkali soil improvement programs [9,10,11,17].

## Data Availability

The dataset supporting the conclusions of this article is available on the NCBI Sequence Read Archive (SRA) platform under the accession number PRJNA1508389.

## Supplementary Materials

The following supporting information can be downloaded at: https://www.mdpi.com/article/10.3390/plants15162480/s1.
